# Editorial: Internalizing disorders in individuals with autism spectrum disorder

**DOI:** 10.3389/fpsyt.2022.1112633

**Published:** 2023-01-09

**Authors:** Valentina Postorino, Luigi Mazzone, Judy Reaven

**Affiliations:** ^1^Departments of Pediatrics and Psychiatry at the University of Colorado, Anschutz Medical Campus, Aurora, CO, United States; ^2^Child Neurology and Psychiatry Unit, Department of Neurosciences, Policlinico Tor Vergata Foundation Hospital, Rome, Italy

**Keywords:** internalizing disorder, autism spectrum disorder (ASD), anxiety, depression, co-occurring conditions in autism

Internalizing disorders are among the most common co-occurring conditions reported in individuals with autism spectrum disorder (ASD) (1–3). Anxiety and depression are the most prevalent internalizing disorders reported in this clinical population. These disorders can exacerbate existing autism symptoms, significantly impact quality of life, and make treatment more challenging. Although these disorders frequently co-occur in autistic individuals, differentiating ASD symptoms from internalizing symptoms can be difficult due to overlap of symptoms between these conditions and/or the fact that autistic symptoms can mask co-occurring mental health symptoms. Furthermore, clarifying the nature of the link between autism and internalizing disorders is currently considered to be a priority in the field, as these disorders can significantly interfere with a child's ability to participate in home, school and community settings, as well as impact on child and family wellbeing.

The research on internalizing disorders in autistic individuals across the lifespan is ongoing and has the potential to significantly improve the lives of people with ASD. In efforts to contribute to the growing literature on this topic, we present a collection of five interesting articles that examine this subject matter from various angles, on the distinguished platform of Frontiers in Psychiatry.

The first article in this Research Topic is a comprehensive systematic review by Hendrix et al. This review summarizes how emotion regulation (ER) has been measured within parent-mediated interventions for young children (at or under the age of 6 years). Furthermore, this paper also describes the extent to which ER is measured concurrently with or distinctly from observable behaviors that have been referenced in existing literature as externalizing or challenging behavior. Results of this review suggest that it is critical to improve measurement of ER within the context of parent-mediated interventions for toddlers and young autistic children.

Emotional dysregulation (ED) is typically expressed with irritability, tantrums, mood fluctuations, and self-harm in young children with autism spectrum disorder (ASD) (4–6). Interventions aimed at improving the ability to recognize and regulate emotions might be an effective way of addressing behavioral problems in this population. Davico et al. investigated the relationship between emotional dysregulation (ED) and adaptive functioning in preschoolers referred for ASD or other neurodevelopmental disorders. Results of this study indicated that ED could represent a specific target for early interventions aimed at enhancing adaptive functioning in early childhood.

Research has shown that autistic adults are at increased risk for current and lifetime depression and suicidality compared to adults without autism (7–9). Some studies have focused on the relationships between negative repetitive thinking and depression in the autistic population. However, little is known about associations between depression and repetitive cognitions and/or behaviors characteristic of autism [i.e., insistence on sameness (IS) and repetitive sensorimotor (RSM) behaviors]. Schwartzman et al. investigated the association between IS, RSM behaviors, and depressive symptoms in a large sample of autistic adults. The results of this study add to the literature on risk factors in the pathway to depression in autism, and offer opportunities for clinical translation regarding screening and intervention efforts.

A growing body of research has shown a higher prevalence of exposure to adverse events and trauma in autistic people compared to non-autistic individuals (10–12). In response to trauma, people use various coping strategies to manage their emotional responses and difficult circumstances. Understanding how people with autism respond to trauma is important to promote recovery in this population. Ng-Cordell et al. present a qualitative study of autistic adults' and caregivers' perspectives on how autistic individuals cope with trauma. Results of this study showed that the utility and accessibility of coping behaviors may depend on personal and contextual factors, including an individual's developmental level, the nature and chronicity of the trauma, and the external resources available to them. Of note, a recurring theme that was called by these authors “Diagnostic Overshadowing” was proposed as impacting the ways in which coping strategies may be misunderstood or overlooked due to the presence of an autism diagnosis.

The final article in this Research Topic tackles the topic of anxiety and dental care. Anxiety related to dental care is elevated in children with ASD, and relatedly, poor dental hygiene and dental problems are also elevated in this population (13, 14). There has been preliminary research in this area, but many questions remain. A manuscript by Park et al. has investigated the association between child-reported dental anxiety and clinical variables expected to be related to dental anxiety across multiple informants (clinicians, parents, and children). The results of this study showed that the majority of participants were estimated to experience clinically significant dental anxiety. This anxiety may lead to refusal to attend dental appointments or difficult-to-manage behavior at the dentist in this population. Therefore, it may be useful to tailor existing treatments to reduce dental anxiety in children with ASD given the long-term consequences this creates.

Internalizing disorders are common in individuals with ASD. These disorders may limit the functioning of autistic individuals across settings (e.g., school, peer relationships) and impact quality of life. This collection of articles helps shed light on the connection between several of these disorders and autism. Results of these papers also highlight the importance of tailored and accessible interventions for this clinical population. Further studies should focus on implementing and disseminating these interventions.

## Author contributions

All authors contributed to the manuscript writing, revision, read, and approved the submitted version.

## References

[1] Hossain MM Khan N Sultana A Ping M McKyer EL Ahmed HU. Prevalence of comorbid psychiatric disorders among people with autism spectrum disorder: an umbrella review of systematic reviews and meta-analyses. Psychiatry Res. (2020) 287:112922. 10.1016/j.psychres.2020.112922 32203749

[2] van Steensel FJ Bögels SM Perrin S. Anxiety disorders in children and adolescents with autistic spectrum disorders: a meta-analysis. Clin Child Fam Psychol Rev. (2011) 14:302–17. 10.1007/s10567-011-0097-0 21735077 PMC3162631

[3] Rast JE Garfield T Roux AM Koffer Miller KH Hund LM Tao S. National Autism Indicators Report: Mental Health. Philadelphia, PA: Life Course Outcomes Program, AJ Drexel Autism Institute, Drexel University (2021).

[4] Lecavalier L. Behavioral and emotional problems in young people with pervasive developmental disorders: relative prevalence, effects of subject characteristics, and empirical classification. J Autism Dev. (2006) 36:1101–14. 10.1007/s10803-006-0147-5 16897387

[5] Samson AC Hardan AY Podell RW Phillips JM Gross JJ. Emotion regulation in children and adolescents with autism spectrum disorder. Autism Res. (2015) 8:9–18. 10.1002/aur.1387 24863869

[6] Mazefsky CA Borue X Day TN Minshew NJ. Emotion regulation patterns in adolescents with high-functioning autism spectrum disorder: comparison to typically developing adolescents and association with psychiatric symptoms. Autism Res. (2014) 7:344–54. 10.1002/aur.1366 24610869 PMC4136477

[7] Cassidy S Bradley P Robinson J Allison C McHugh M Baron-Cohen S. Suicidal ideation and suicide plans or attempts in adults with asperger's syndrome attending a specialist diagnostic clinic: a clinical cohort study. Lancet Psychiat. (2014) 1:142–7. 10.1016/S2215-0366(14)70248-2 26360578

[8] Hudson CC Hall L Harkness KL. Prevalence of depressive disorders in individuals with autism spectrum disorder: a meta-analysis. J Abnorm Child Psychol. (2019) 47:165–75. 10.1007/s10802-018-0402-1 29497980

[9] Hollocks MJ Lerh JW Magiati I Meiser-Stedman R Brugha TS. Anxiety and depression in adults with autism spectrum disorder: a systematic review and meta-analysis. Psychol Med. (2019) 49:559–72. 10.1017/S0033291718002283 30178724

[10] Hoover DW Kaufman J. Adverse childhood experiences in children with autism spectrum disorder. Curr Opin Psychiatry. (2018) 31:128. 10.1097/YCO.0000000000000390 29206686 PMC6082373

[11] Kerns CM Newschaffer CJ Berkowitz S Lee BK. Brief report: examining, the association of autism and adverse childhood experiences in the National Survey of, Children's Health: the important role of income and co-occurring mental health, conditions. J Autism Dev Disord. (2017) 47:2275–81. 10.1007/s10803-017-3111-7 28378271 PMC5523127

[12] McDonnell CG Boan AD Bradley CC Seay KD Charles JM Carpenter LA. Child maltreatment in autism spectrum disorder and intellectual disability: results from a population-based sample. J Child Psychol Psych. (2019) 60:576–84. 10.1111/jcpp.12993 30368827 PMC6458088

[13] Sahab LA. Investigating Dental Anxiety in Individuals With Autism Spectrum Disorders (PhD thesis Reading). University of Reading (2017).

[14] Elmore JL Bruhn AM Bobzien JL. Interventions for the reduction of dental anxiety and corresponding behavioral deficits in children with autism spectrum disorder. Am Dental Hygien Associat. (2016) 90:111–20. 27105789

